# European Recommendations for Transitioning the Care of Patients With Multiple Myeloma Treated With B‐Cell Maturation Antigen Bispecific Antibodies From Academic Hospitals to Community‐Based Centers and for Outpatient Step‐Up Dosing

**DOI:** 10.1002/jha2.70290

**Published:** 2026-05-14

**Authors:** María‐Victoria Mateos, Elena Zamagni, Massimo Gentile, Rachel Hall, Florence Lachenal, Mónica López Riñon, Rakesh Popat, Christof Scheid, Thomas Wolff, Isabel Perez‐Cruz, Mohamad Mohty

**Affiliations:** ^1^ University Hospital of Salamanca/IBSAL/Cancer Research Center–IBMCC (USAL‐CSIC) Salamanca Spain; ^2^ Istituto Di Ematologia “Seràgnoli”, IRCCS Azienda Ospedaliero‐Universitaria Di Bologna Bologna Italy; ^3^ Dipartimento Di Scienze Mediche E Chirurgiche Università Di Bologna Bologna Italy; ^4^ Hematology Unit, Department of Onco‐Hematology A.O. of Cosenza Cosenza Italy; ^5^ Department of Pharmacy, Health and Nutritional Science University of Calabria Rende Italy; ^6^ University Hospitals Dorset NHS Foundation Trust Bournemouth UK; ^7^ Groupement Hospitalier Nord‐Dauphiné (GHND) Bourgoin‐Jallieu France; ^8^ Hospital General La Mancha Centro, Alcázar de San Juan Ciudad Real Spain; ^9^ Hospital General de Tomelloso Tomelloso Spain; ^10^ University College London Hospitals NHS Foundation Trust London UK; ^11^ Cologne University Hospital Cologne Germany; ^12^ Onkologie Lerchenfeld Hamburg Germany; ^13^ Pfizer Inc. New York New York USA; ^14^ Hôpital Saint Antoinel Sorbonne University Paris France; ^15^ NSERM UMRs938 Paris France

**Keywords:** BCMA, bispecific antibodies, consensus, multiple myeloma, outpatient, step‐up dosing

## Abstract

**Introduction:**

Safe outpatient delivery of B‐cell maturation antigen–targeting bispecific antibodies (BCMA‐BsAbs) in multiple myeloma (MM) is challenging, particularly during step‐up dosing (SUD). Standardized guidance for European community practice is limited.

**Methods:**

A modified Delphi methodology panel sought expert consensus on safe, effective outpatient SUD and transition of care from academic to community centers for patients receiving BCMA‐BsAbs. A steering committee of 10 European specialists in MM and BCMA‐BsAb delivery generated statements based on literature review and clinical experience. Statements were tested in three rounds with a Delphi panel of 53 clinicians (hematologists, nurses, pharmacists) across France, Germany, Italy, Portugal, Spain, and the United Kingdom. Consensus was defined as ≥ 75% agreement.

**Results:**

Consensus was achieved for 84 statements. Key recommendations included: (1) patient stability before community center transition (afebrile ≥ 24 h, ASTCT Grade 0 toxicity, stable organ function); (2) community center requirements (training in cytokine release syndrome/immune effector cell–associated neurotoxicity syndrome, urgent laboratory access, tocilizumab and corticosteroids on‐site, 24/7 escalation protocols); and (3) infection management (baseline cytomegalovirus testing, immunoglobulin [Ig] monitoring, prophylaxis with intravenous/subcutaneous Ig for IgG < 400 mg/dL, antiviral/*Pneumocystis jirovecii* pneumonia prophylaxis). Outpatient SUD should be restricted to clinically stable patients with low tumor burden and reliable caregiver support and conducted only in centers with staff trained in BCMA‐BsAb toxicity management and a clear escalation plan.

**Conclusion:**

This European Delphi consensus provides a structured framework for outpatient SUD and transition of BCMA‐BsAb therapy in MM. Adopting these recommendations may improve safety and consistency and expand accessibility.

Trial Registration: The authors have confirmed clinical trial registration is not needed for this submission

## 1 Introduction

Despite therapeutic advances, multiple myeloma (MM) remains incurable for the majority of patients, with most experiencing multiple relapses and later lines of therapy characterized by shorter response durations and increased toxicity [1, 2].

The emergence of T‐cell–redirecting therapies, particularly B‐cell maturation antigen–targeting bispecific antibodies (BCMA‐BsAbs), offers new hope for heavily pretreated patients [3]. The two BCMA‐BsAbs approved for subcutaneous administration, elranatamab and teclistamab [4, 5], have demonstrated deep and durable responses, even in triple‐class refractory disease [6, 7]. Nevertheless, these BsAbs present challenges, including potential adverse effects such as the risk of cytokine release syndrome (CRS), immune effector cell–associated neurotoxicity syndrome (ICANS), hypogammaglobulinemia, and infection, which require careful monitoring and appropriate management [8]. Step‐up dosing (SUD) protocols and infection prophylaxis are critical to mitigating these risks [3, 8], but implementation requires robust coordination across care settings to improve the current use of healthcare system resources.

Initial BCMA‐BsAb doses are often administered in academic centers/university hospitals with intensive monitoring by staff trained and familiar with adverse event monitoring and management [3]. This is followed, in some cases, by transition to the community care setting (community hospitals, outpatient clinics, or physician offices). However, transition and outpatient SUD protocols lack robust, standardized frameworks to determine patient eligibility, minimum infrastructure, handover procedures, and patient/caregiver education regarding outpatient and community‐setting delivery of BCMA‐BsAbs in MM.

Given the growing MM patient population due to population aging and advancements in diagnostic capabilities [9, 10, 11], the rapid uptake of BCMA‐BsAbs [12, 13, 14], and an increased desire to provide these therapies in the community setting, there is an urgent need to define best practices for outpatient provision of these agents. The Delphi consensus method is very well suited to this challenge, offering a systematic approach to synthesizing expert opinion where empirical evidence is limited. This manuscript presents consensus recommendations to guide transitions of care from university/academic centers to community clinics and support safe, effective, and accessible outpatient delivery of BCMA‐BsAbs to patients with MM across Europe.

## 2 Methods

**FIGURE 1:**
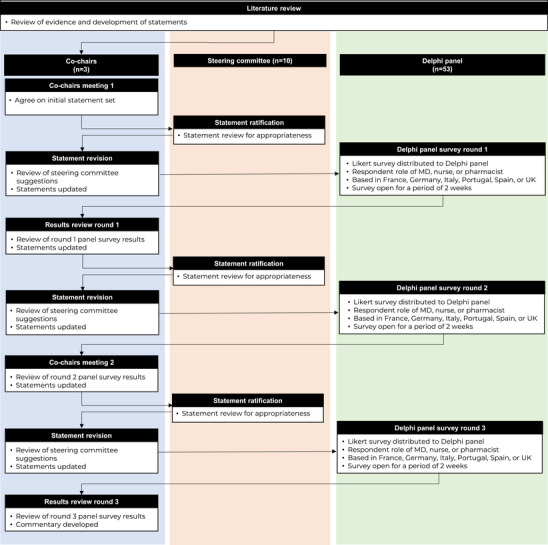
Modified Delphi study design. MD indicates physician.

The process followed a modified Delphi methodology (Figure 1) involving three co‐chairs (Drs. Mateos, Mohty, and Zamagni) working within a wider steering committee containing seven additional physicians with recognized expertise in the treatment of MM and delivery of subcutaneous BCMA‐BsAbs from the target countries. The steering committee members were from the United Kingdom, Spain, Germany, Italy, and France, representing five academics and five community MM practices. Once the statements were agreed upon by the steering committee, they were tested in three rounds of survey with a wider Delphi panel (*n* = 53).

The panel included the steering committee and additional experts (physicians, nurses, and pharmacists) who were recruited based on individual clinical experience with MM (including the use of BCMA‐BsAbs) from both academic and nonacademic centers based in France, Germany, Italy, Spain, Portugal, and the United Kingdom. Recruitment continued during the consensus process, resulting in 40, 39, and 53 panelists for Rounds 1, 2, and 3, respectively. All were required to provide biographical information to establish appropriateness for participation.

The initial research question was, what is the optimal way to transition from academic to community settings when using MM BsAb therapy, and what best practice exists for outpatient SUD? In June 2025, a targeted review of published and publicly available data on transitions of care from academia to community settings for patients with MM treated with BCMA‐BsAbs and on outpatient SUD of BsAb therapy was conducted using PubMed and Google. Seventy‐one papers were selected as relevant to this review. The facilitator (Triducive Partners Ltd.) used these findings to develop an initial set of consensus statements for discussion during the first co‐chairs meeting.

Following the meeting, the statement set was incorporated into a survey using Microsoft Forms. This was sent to the steering committee for ratification over two rounds (July–September 2025), and each statement offered the options of Accept, Reject, or a free‐text field for suggested changes. The results were analyzed and revised by the co‐chairs, as required. A majority of votes was required to trigger statement deletion, but suggested changes were included if they were considered by the co‐chairs to be appropriate.

Sixty‐nine statements were agreed upon for testing with the Delphi panel. The panel survey presented each statement along with a four‐point Likert response scale (*strongly agree*, *tend to agree*, *tend to disagree*, and *strongly disagree*) for respondents to indicate their corresponding level of agreement. The identity of the panel respondents was known to the facilitator but not reported to the co‐chairs or steering committee.

The survey captured data including respondent country (France, Germany, Italy, Portugal, Spain, and the United Kingdom), role (MD, nurse, pharmacist), and number of years of experience treating MM (≤ 5, 6–10, 11–20, ≥ 21 years). The survey was written in English. The survey link was distributed by the independent facilitator to the panel via email and a reminder sent after 1 week. All responses were included in the analysis for each round.

After each round, the responses were aggregated to provide an overall agreement level (i.e., the number of respondents expressing agreement as a percentage of the overall number of responses for each statement). After each round, a briefing of the results was sent to the co‐chairs and a virtual meeting was held for comment (on statements that did not reach consensus) and suggested actions (removal or refinement of existing statements, or addition of new statements). Follow‐up was provided by email to co‐chairs who were unable to join virtually. At the end of survey Round 2, the second co‐chairs meeting was convened to discuss the current statement set ahead of the third and final round.

All suggested new and amended statements were sent to the steering committee for ratification prior to surveying the panel. Due to low response rates and delays with institutional approvals for some German community physicians, it was agreed to seek further responses for Round 3 to gain a more representative sample. A third party (M3 Global Research, an agency that maintains a curated portfolio of MM physicians) was engaged to obtain additional responses, resulting in a further 12 responses from Germany for Round 3 (September–October 2025).

Three rounds of panel survey were agreed upon as the stopping criteria for the consensus process, and a threshold for consensus was set at 75% (a widely accepted threshold) [15].

A statement of consent was included at the start of the survey, and consent was implied by completion. As this study collected only the opinions of healthcare professionals and no patient‐specific data were captured, ethical approval was not sought. This study was not prospectively registered.

Presentation of the study findings adhered to ACCORD (ACcurate COnsensus Reporting Document) reporting guidelines (see Supporting Information) [16].

## 3 Results

### 3.1 Panel Round 1

In Round 1, 38 completed surveys were received (response rate, 95%). Respondent role, years of experience in treating MM, and country for all three rounds are shown in Table 1.

**TABLE 1:** Respondent demographics according to panel survey Rounds 1–3.

	Round 1	Round 2	Round 3
Role, *n*			
MD	26	26	34
Nurse	6	6	9
Pharmacist	6	7	10
Country, *n*			
France	5	6	5
Germany	3	4	17
Italy	9	11	12
Portugal	2	0	2
Spain	11	11	11
United Kingdom	8	7	6
Experience, *n*			
≤ 5 years	1	3	2
6–10 years	7	6	10
11–20 years	14	18	24
≥ 21 years	16	12	17

*Note*: MD indicates physician.

Results from Round 1 demonstrated consensus (≥ 75%) for 68/69 statements, of which 52 (75%) achieved ≥ 90% agreement. One statement failed to achieve consensus. The steering committee reviewed the total statement set and agreed to refine and reissue 26 statements for retesting in panel Round 2.

### 3.2 Panel Round 2

At the end of the second round, 39 responses were received from the panel (Table 1). Of the 26 revised statements, 22 achieved consensus, with 19 (73%) achieving ≥ 90% agreement; four statements did not achieve consensus. The response rate for Round 2 was 100%. The steering committee reviewed the Round 2 statement set and agreed to refine and reissue three statements and add a further five for testing in panel Round 3.

### 3.3 Panel Round 3

At the end of the third round, 53 responses were received from the panel (41 from the previous panel rounds and an additional 12 specifically recruited from Germany to provide better representation). All eight statements achieved consensus, one achieving ≥ 90% agreement. The response rate for Round 3 was 100%. The final list of statements achieving consensus is shown in Tables 2 and 3.

**TABLE 2:** Final statements on the transition of care from academic to community settings achieving consensus over the three panel rounds.

		Round tested	Agreement
**Key patient criteria**			
1.	Patients must be afebrile (≥ 24 h) and hemodynamically stable before being considered for transition to community care.	2	97%
2.	Patients must be ASTCT Grade 0 (no toxicity) before being considered for transition to community care.	2	95%
3.	Patients must demonstrate stable hematologic, renal, and hepatic function, with no new or worsening cytopenias (e.g., neutropenia, thrombocytopenia), renal impairment, or transaminitis.	1	90%
4.	Patients should not be transitioned unless they have completed the full SUD phase and any occurring CRS or ICANS has resolved, except if they have not had CRS or ICANS with the first two doses.	1	90%
**Key community center criteria**			
5.	Community centers delivering BCMA‐BsAbs should be trained by clinical leads and resourced to manage CRS and ICANS using ASTCT and IMWG grading systems.	1	97%
6.	Community centers delivering BCMA‐BsAbs should have documented escalation protocols outlining how to formally contact the academic center, including procedures for 24‐h communication, in case support or intervention is needed.	1	90%
7.	Community centers delivering BCMA‐BsAbs should have on‐site access to routine urgent blood tests (e.g., FBC, CRP, LFTs, renal function).	1	97%
8.	Community centers delivering BCMA‐BsAbs should have 24/7 access to tocilizumab and corticosteroids (e.g., dexamethasone) for rapid CRS or ICANS intervention and trained personnel to administer them.	1	95%
9.	Community centers delivering BCMA‐BsAbs should be located within 60 min (by ambulance, car, etc.) of a facility with access to inpatient facilities.	1	97%
**Infection and Ig management**			
10.	All patients should undergo baseline assessment for CMV (serology followed by PCR if serology is positive for CMV) to allow for accurate assessment of subsequent reactivation.	1	92%
11.	Prophylactic IV/SC Ig prior to the first dose of BsAb therapy in multiple myeloma should be considered on a case‐by‐case basis for ***all*** patients (e.g., taking IgG levels and BsAb type into account).	2	82%
12.	Prophylactic IV/SC Ig prior to the first dose of BsAb therapy in multiple myeloma should be considered for all patients with IgG < 400 mg/dL.	2	95%
13.	Prophylactic IV/SC Ig prior to the first dose of BsAb therapy in multiple myeloma should be considered for all patients with recurrent infections.	2	89%
14.	Prophylactic IV/SC Ig should be initiated early and maintained in patients with IgG < 400 mg/dL or with recurrent infections.	2	97%
15.	Patients with immunoparesis irrespective of the target IgG should receive IV/SC Ig, particularly if there is a risk of recurrent or severe infections.	1	84%
16.	Patients with trough IgG < 400 mg/dL should receive IV or SC Ig every 4 weeks and be retested every month until IgG recovers (including after completion of treatment).	1	95%
17.	Neutropenia (defined as an absolute neutrophil count < 1000/mm^3^) should not be resolved with G‐CSF prior to SUD due to the risk of CRS.	3	79%
18.	Neutropenia (defined as an absolute neutrophil count < 1000/mm^3^) caused by high tumor burden increases the risk of CRS and should not be resolved with G‐CSF prior to SUD.	3	79%
19.	Baseline and monitoring of serum IgG every 4 weeks should be conducted, with timely access to IV/SC Ig as clinically indicated.	1	100%
20.	SC Ig may be considered if available to reduce hospital administrative burden while maintaining immunoprophylaxis.	1	95%
21.	Antiviral (e.g., acyclovir) and PJP prophylaxis (e.g., co‐trimoxazole) are mandatory for all patients.	1	100%
22.	Antifungal (e.g., posaconazole/fluconazole) and antibacterial prescriptions should be tailored to individual risk factors, prior lines of therapy, and lymphodepletion status.	1	100%
**Handover process and documentation**			
23.	Academic centers should provide 3–5 days’ notice before transitioning care to the community setting.	1	84%
24.	A comprehensive treatment summary, including BsAb dosing history, CRS/ICANS grading, and infection prophylaxis, and all AEs and management must be provided at handover.	1	100%
25.	Patients and care partners must receive education on early signs/symptoms of CRS, ICANS, and infection (e.g., fever, confusion, respiratory symptoms) at discharge.	1	100%
26.	Follow‐up with academic centers via telemedicine, a virtual meeting, or a phone call should be scheduled within 1 week of transition.	1	76%
**Patient education and support**			
27.	Patients and care partners should receive a 24/7 emergency contact and escalation plan before transition.	1	97%
28.	Education on CRS/ICANS must include both verbal explanations and written materials tailored to BCMA‐BsAb toxicities.	1	100%
29.	Language‐appropriate educational materials must be provided to patients and care partners in their first language.	1	100%

Abbreviations: AE: adverse event; ASTCT: American Society for Transplantation and Cellular Therapy; BCMA: B‐cell maturation antigen; BsAb: bispecific antibody; CMV: cytomegalovirus; CRP: C‐reactive protein; CRS: cytokine release syndrome; FBC: full blood count; G‐CSF: granulocyte colony‐stimulating factor; ICANS: immune effector cell–associated neurotoxicity syndrome; Ig: immunoglobulin; IMWG: International Myeloma Working Group; IV: intravenous; LFT: liver function test; PCR: polymerase chain reaction; PJP: *Pneumocystis jirovecii* pneumonia; SC: subcutaneous; SUD: step‐up dosing.

**TABLE 3:** Final statements on outpatient step‐up dosing achieving consensus over the three panel rounds.

		Round tested	Agreement
**Key patient criteria**			
30.	Outpatient SUD should only be considered for patients with ECOG performance status of 0, 1, or 2.	1	92%
31.	Outpatient SUD should only be considered for patients with low tumor burden (where high tumor burden is defined as BMPCs ≥ 60%, presence of ≥ 1 focal lesion ≥ 5 mm [bone or soft tissue] on MRI or PET–CT, M‐spike ≥ 30 g/L, LDH > ULN) and stable disease status.	1	84%
32.	Outpatient SUD should only be considered for patients with lytic bone lesions if potential complications (e.g., fracture risk, spinal cord compression) have been evaluated and appropriate monitoring and support measures are in place.	1	87%
33.	Outpatient SUD should only be considered for patients with renal impairment or failure, including chronic kidney disease at any stage, if renal function is stable enough to support safe outpatient management.	1	87%
34.	Outpatient SUD should only be considered for patients with ESRD providing they are clinically stable and have reliable access to dialysis, and structured pathways are in place for toxicity monitoring and escalation.	1	82%
35.	Outpatient SUD should only be considered for patients with hypertension if it is well controlled and not associated with additional cardiovascular risks.	1	100%
36.	Outpatient SUD should only be considered for patients with congestive heart failure if they are clinically stable (e.g., NYHA Class I/II), have no recent decompensation, and have undergone appropriate cardiac evaluation.	1	95%
37.	Outpatient SUD should only be considered for patients with chronic pulmonary disease if respiratory status is stable and oxygenation needs are met (e.g., FEV_1_ > 50%).	2	89%
38.	Outpatient SUD should only be considered for patients with a history of myocardial infarction if they have preserved cardiac function (LVEF of ≥ 45%), are clinically stable, and are not at high risk for further cardiovascular events.	1	100%
39.	Outpatient SUD should generally not be offered to patients with Grade 3/4 neutropenia unless in exceptional, carefully monitored settings. A minimum ANC ≥ 1000/mm^3^ is generally recommended.	2	95%
40.	Outpatient SUD should only be considered for patients with no neurological symptoms (e.g., cognitive impairment, focal neurological deficits, or seizure risk factors).	1	100%
41.	Outpatient SUD should only be considered for patients with cerebrovascular disease if they are neurologically stable and not at high risk for acute events.	1	90%
42.	Outpatient SUD should only be considered for patients with peripheral vascular disease if it is not associated with high thrombotic risk during treatment.	1	90%
43.	Outpatient SUD should only be considered for patients with diabetes if it is well managed and without acute complications.	1	95%
44.	Outpatient SUD should only be considered for patients with hypercalcemia if it is corrected and stable before SUD initiation.	1	92%
45.	Outpatient SUD should only be considered for patients with no active infection (e.g., CMV reactivation, upper respiratory tract infection).	1	97%
46.	Patients must reside within 60 min (by ambulance, car, etc.) of a treatment center that provides both hematology or internal medicine expertise and ICU‐level care.	1	100%
47.	Patients must have a suitable adult care partner available 24/7 during the SUD period who has received CRS/ICANS training.	1	100%
48.	Patients must have a responsible adult care partner available 24/7 during the SUD period who is physically and cognitively able to support the patient and understands when to seek urgent care.	1	100%
49.	The center/provider that provided CRS/ICANS training for the care partner should be documented by the trainer and a copy retained in the patient notes, if required.	1	95%
**Key center criteria and protocols**			
50.	Outpatient SUD should only be delivered in centers that can coordinate access to an ICU.	1	95%
51.	Outpatient SUD centers must ensure availability of a clinician (e.g., hematologist or trained advanced clinical practitioner) experienced in managing CRS and ICANS during dosing hours (e.g., experienced in use of BsAbs and able to supervise outpatient SUD).	1	97%
52.	Outpatient SUD centers must have tocilizumab (two doses) available in a known and accessible location.	1	97%
53.	Outpatient SUD centers must have access to dexamethasone, high‐dose corticosteroids, and anakinra for management of emergent CRS or ICANS.	1	97%
54.	Outpatient SUD centers must have access to oral valganciclovir or letermovir or alternatively intravenous ganciclovir or foscarnet for treatment of reactivated or emergent CMV.	1	97%
55.	SOPs must define observation duration and escalation thresholds for CRS and ICANS, using established grading systems such as ASTCT and ICE.	1	100%
56.	Emergency care must be accessible within 30–60 min of the outpatient SUD center and must provide fast‐track access protocol, inpatient admission capability, access to intensive care, and direct communication with the treating hematology team.	1	97%
57.	Following each step‐up dose, patients must be observed for vital signs and neurological function for a minimum defined period as specified in the SmPC.	1	100%
58.	Standardized admission protocols aligned with the SmPC are required for outpatient SUD.	1	100%
59.	A formal protocol should be developed to guide reescalation of care to the academic center for patients experiencing clinical deterioration following transfer to a community site after BsAb therapy initiation. This protocol should integrate rapid‐response triggers based on clinical efficacy, safety indicators, and/or patient‐centered factors (e.g., quality of life, goals of care), with flexibility for urgent transfers and adaptation to local resources.	2	92%
**Defining high‐risk patients**			
60.	Biological high‐risk disease can be defined as the presence of ≥1 of the following: At least 1 of (a) del(17p), with a cutoff of >20% clonal fraction, and/or TP53 mutation; (b) an IgH translocation including t(4;14), t(14;16), or t(14;20) along with 1q+ and/or del(1p32); (c) monoallelic del(1p32) along with 1q+ or biallelic del(1p32); or (d) β_2_‐microglobulin ≥5.5 mg/L with normal creatinine (< 1.2 mg/dL) ISS Stage III disease Elevated LDH ≥ 223 U/L Extramedullary plasmacytomas Plasma cell leukemia Validated high‐risk gene expression profile	2	93%
61.	Clinical high‐risk disease can be defined as ≥ 1 of the following: Cardiovascular disease (especially heart failure) Poorly controlled diabetes COPD Severe chronic kidney disease Dementia	1	97%
62.	Systematic AE reporting processes using standardized criteria (e.g., CTCAE) should be in place for all patients receiving BsAbs, regardless of the treatment setting, and should be simple, practical, and integrated into routine workflows.	2	95%
63.	CMV infection/reactivation should not be routinely monitored during SUD unless febrile illness occurs without bacterial cause.	1	100%
64.	In suspected CMV infection/reactivation, BsAb therapy should be placed on hold, with active monitoring of the viral load.	1	100%
65.	Premedications (e.g., dexamethasone, antihistamines, paracetamol) must be administered before each step‐up dose in accordance with the SmPC.	1	97%
66.	Neurotoxicity should be assessed and documented using both ICE and ASTCT criteria during SUD and at each subsequent visit.	2	97%
67.	Toxicity data should be collected through the first full treatment dose and beyond 14 days.	2	95%
68.	CRS should be assessed and documented using ASTCT CRS criteria.	1	100%
69.	Remote monitoring tools (e.g., check‐in calls) may be useful to support patients during outpatient SUD.	1	100%
70.	Patients undergoing SUD should receive remote daily safety calls from a member of the medical team, except on days when they are seen in person at the hospital or clinic.	2	92%
71.	Protocols should be established to enable immediate admission to the treating center for prompt management of toxicities.	1	100%
72.	Prophylactic tocilizumab could be considered prior to the first step‐up dose in patients to reduce the risk of CRS, where appropriate and available.	3	77%
73.	A single IV 8‐mg/kg dose of tocilizumab (max dose of 800 mg) could be administered as a pretreatment on C1D1 prior to starting administration of BCMA‐BsAb therapy for outpatient SUD, where appropriate and available.	3	79%
74.	In the absence of tocilizumab prophylaxis, prophylactic dexamethasone (12 mg) should be considered on D2 of SUD.	2	76%
75.	In the absence of tocilizumab prophylaxis, the use of prophylactic dexamethasone (12 mg) could be considered as part of step‐up dosing in addition to the dexamethasone recommended on the product label.	3	81%
76.	Oral dexamethasone (10–20 mg) can be used only under direct instruction from a treating physician when CRS or ICANS is suspected, en route to the hospital, or as a strategy for at‐home management of Grade 1 CRS or ICANS. Clear criteria and escalation instructions must be provided to patients and care partners along with education regarding the risks of inappropriate use.	1	92%
77.	Community centers that cannot initiate these medicines could refer to academic centers for step‐up dosing.	3	94%
78.	BCMA‐BsAb products should be prepared in a safety cabinet to maintain aseptic conditions.	3	89%
**Logistics and follow‐up**			
79.	A minimum interval of 48 h must be maintained between step‐up doses, as per the SmPC.	1	100%
80.	Given the typical onset of CRS/ICANS within 1–3 days of dosing, patients should undergo a clinical review, either in person or virtually, within 24–72 h of completing the SUD schedule.	1	100%
81.	Outpatients should have a normal ICE score before leaving the treatment facility following the final step‐up dose.	1	100%
**Education and communication**			
82.	All clinical and pharmacy staff supporting SUD should be trained in CRS/ICANS and infection recognition (including opportunistic), grading, and emergency management.	2	100%
83.	Staff at community centers administering BsAb therapy should undergo simple, practical, structured training (e.g., using quick‐reference materials), as defined by a standardized protocol. This training should cover key topics such as CRS/ICANS management, patient monitoring, and emergency escalation procedures, and their completion documented appropriately.	2	100%
84.	Patients and care partners should receive clear verbal and written instructions on recognizing complications and how to access urgent care pathways.	1	97%

Abbreviations: AE: adverse event; ANC: absolute neutrophil count; ASTCT: American Society for Transplantation and Cellular Therapy; BCMA: B‐cell maturation antigen; BMPC: bone marrow plasma cell; BsAb: bispecific antibody; C: cycle; CMV: cytomegalovirus; COPD: chronic obstructive pulmonary disease; CRS: cytokine release syndrome; CTCAE: Common Terminology Criteria for Adverse Events; D: day; ECOG: Eastern Cooperative Oncology Group; ESRD: end‐stage renal disease; FEV_1_: forced expiratory volume in 1 s; ICANS: immune effector cell–associated neurotoxicity syndrome; ICE: immune effector cell–associated encephalopathy; ICU: intensive care unit; Ig: immunoglobulin; IgH: immunoglobulin heavy chain; ISS: International Staging System; IV: intravenous; LDH: lactate dehydrogenase; LVEF: left ventricular ejection fraction; MRI: magnetic resonance imaging; NYHA: New York Heart Association; PET–CT: positron emission tomography–computed tomography; SmPC: summary of product characteristics; SOP: standard operating procedure; SUD: step‐up dosing; TP53: tumor protein p53; ULN: upper limit of normal.

Over the three rounds, 97/103 statements (94%) achieved consensus, resulting in a final list of 84 recommended standalone criteria for community and academic centers regarding the transition of care, designed to support healthcare providers in setting up an ambulatory treatment service (including outpatient SUD) for the use of BCMA‐BsAbs in MM.

During the panel Round 1 survey, only one statement (concerning the prophylactic use of tocilizumab prior to the first step‐up dose; Table S1) failed to achieve consensus. The co‐chairs wished to test this lack of consensus further and in Round 2 included two statements concerning prophylactic tocilizumab, both of which failed to achieve consensus (Table S2). These statements were revised again and retested in Round 3, in which they finally achieved consensus.

Regarding patterns of response, no specific patterns of variation were identified between respondent countries, roles, or years of experience in MM, but this may have been hampered by variable representation between subgroups.

**FIGURE 2:**
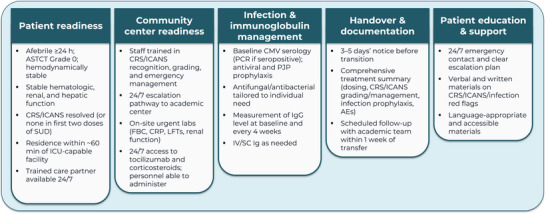
Operational requirements for transfer of care from academic to community centers. AE: adverse event; ASTCT: American Society for Transplantation and Cellular Therapy; CMV: cytomegalovirus; CRP: C‐reactive protein; CRS: cytokine release syndrome; FBC: full blood count; h: hours; ICANS: immune effector cell–associated neurotoxicity syndrome; ICU: intensive care unit; Ig: immunoglobulin; IV: intravenous; LFT: liver function test; min: minutes; PCR: polymerase chain reaction; PJP: *Pneumocystis jirovecii* pneumonia; SC: subcutaneous; SUD: step‐up dosing.

**FIGURE 3:**
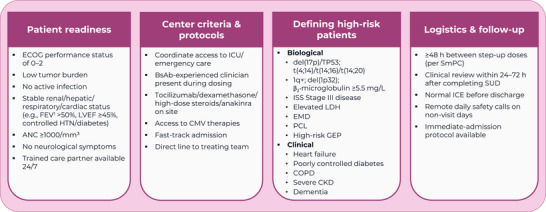
Operational requirements for outpatient step‑up dosing. ANC: absolute neutrophil count; BsAb: bispecific antibody; CKD: chronic kidney disease; CMV: cytomegalovirus; COPD: chronic obstructive pulmonary disease; ECOG: Eastern Cooperative Oncology Group; EMD: extramedullary disease; FEV_1_: forced expiratory volume in 1 s; GEP: gene expression profile; h: hours; HTN: hypertension; ICE: immune effector cell–associated encephalopathy; ICU: intensive care unit; ISS: International Staging System; LDH: lactate dehydrogenase; LVEF: left ventricular ejection fraction; PCL: plasma cell leukemia; SmPC: summary of product characteristics; SUD: step‐up dosing; TP53: tumor protein p53.

The consensus results were used to develop a set of checklists for centers to use to support community care transfer and/or outpatient SUD (Supporting Information). A summary of the agreed‐upon operational requirements is shown in Figures 2 and 3.

## 4 Discussion

BCMA‐BsAbs represent a transformative step in the management of relapsed/refractory MM, produce deep responses, and offer a rapid, off‐the‐shelf treatment option for patients [6, 7]. These therapies, like other T‐cell–engaging therapies, are associated with CRS and ICANS. Furthermore, cytopenias, hypogammaglobulinemia, and increased infection risk are hallmarks of MM treatment. Recommendations for reducing the risk of and controlling these adverse events are essential to ensure safe and sustainable integration of BCMA‐BsAbs into both community settings and outpatient models of care in Europe [8, 17].

The Delphi process achieved high agreement (nearly 80% of the 84 final statements achieved ≥ 90% agreement), reflecting strong convergence among European experts regarding safety recommendations to support a safe transition of care from academic to community centers and outpatient SUD. The resulting framework expands upon the earlier European Myeloma Network [8] and International Myeloma Working Group (IMWG) [3] recommendations by incorporating operational and educational standards that directly address challenges implementing outpatient care, including staff competencies, community center criteria (training, medication access, protocols, resource requirements), patient selection criteria, infection prophylaxis, remote monitoring, and interinstitutional communications.

### 4.1 Transition of Care From Academic to Community Settings

Results emphasized that only clinically stable patients (e.g., those afebrile, free of CRS/ICANS, and with preserved organ function) should transition from academic centers to community sites.

A key strength of this consensus is the delineation of infrastructure requirements for community centers. Respondents highlighted the necessity of trained staff competent in CRS/ICANS recognition and management, 24/7 availability of tocilizumab and corticosteroids, urgent laboratory access, and formalized escalation protocols. Embedding these safeguards may mitigate risk as administration of BCMA‐BsAbs shifts closer to the community setting [18].

The immunosuppressive effects of BCMA‐BsAbs necessitate proactive infection prevention and management. The panel endorsed baseline cytomegalovirus testing, routine immunoglobulin (Ig) monitoring, and tailoring antibacterial and antifungal prescriptions to individual risk factors, prior lines of therapy, and lymphodepletion status. The panel also endorsed the prophylactic use of intravenous/subcutaneous IgG. Importantly, 97% of panelists agreed that prophylactic IgG therapy should be initiated and maintained in patients with IgG < 400 mg/dL or recurrent infections, and 82% agreed that all patients (regardless of IgG levels) should be considered for IgG therapy prior to the first BCMA‐BsAb dose. These recommendations reflect evidence reporting improvements in progression‐free and overall survival [19] and a 90% reduction in Grade ≥ 3 infections [20, 21, 22]. Antiviral and *Pneumocystis jirovecii* prophylaxes were both considered mandatory, reflecting current IMWG guidelines, European Myeloma Network consensus, and expert recommendations [3, 8, 17]. Standardizing immunoprophylaxis protocols may therefore reduce morbidity and associated healthcare utilization.

Given intra‐ and inter‐country variability in transition processes, robust communication between academic and community centers is needed to maximize safety. The panel endorsed comprehensive treatment summaries, sufficient notice before transition, early patient and caregiver education, a 24/7 emergency contact and clear escalation plan, and follow‐up with academic teams within 1 week of transfer. Such hybrid care models, combining decentralized treatment with central oversight, may represent a pragmatic solution for Europe's heterogeneous healthcare systems [14].

### 4.2 Outpatient SUD and Monitoring

The consensus aims to support outpatient SUD by providing clear guidance on patient eligibility (Eastern Cooperative Oncology Group performance status of 0–2, low tumor burden, stable organ function, reliable caregiver) and center capabilities (trained staff, rapid escalation pathways, intensive care unit coordination). Importantly, the panel agreed that outpatient SUD is feasible only when strict patient, caregiver, and institutional prerequisites are met. These include residence within 60 min of an inpatient facility, presence of a trained adult caregiver 24/7, and access to urgent escalation pathways. These parameters align with other findings [14] and suggest that outpatient BCMA‐BsAb dosing is achievable when risk‐adapted protocols and robust follow‐up are in place.

### 4.3 High‐Risk Disease

The co‐chairs and steering committee discussed that in addition to the new IMWG definition of high‐risk disease [23], other biological indicators should be considered as they are associated with more aggressive disease, treatment resistance, and poorer prognosis [24, 25]. In addition to the updated IMWG high‐risk criteria [23], Statement 60 (“biological high‐risk disease”) includes elevated lactate dehydrogenase level, International Staging System Stage III disease, extramedullary plasmacytomas, plasma cell leukemia, and a validated high‐risk gene expression profile. Guidance is also provided for comorbidity‐driven risk stratification (Statement 61: “clinical high‐risk disease”). While none of these factors are contraindications to outpatient SUD, the recommendations assist practical decisions regarding outpatient SUD eligibility.

### 4.4 Cytokine Release Syndrome

Incidence, severity, and timing of CRS are also important considerations when transitioning care from the academic to community setting and for outpatient SUD. CRS events with elranatamab (57.9% overall) were primarily low‐grade (43.7%, 13.7%, and 0.5% for Grades 1, 2, and 3, respectively) and were mainly confined to SUD (43.2% and 19.1% after the first and second SUD, respectively), with 7.1% and 1.6% of patients experiencing CRS after the first full treatment dose and subsequent treatment doses, respectively [4]. With teclistamab, CRS events were 72% overall (50%, 21%, and 0.6% for Grades 1, 2, and 3, respectively), with 44% and 35% occurring after the first and second SUD, respectively, and 24% and < 3% after the initial maintenance dose and subsequent doses, respectively [5]. With both elranatamab and teclistamab, the median time to onset of CRS was 2 days after the most recent dose and the median duration was 2 days.

The integration of daily remote safety calls and telemedicine oversight reflects a growing trend toward hybrid care models. Indeed, remote vital‐sign tracking and daily symptom calls have been used successfully to detect early CRS or infection events with elranatamab and teclistamab [26, 27, 28]. These digital strategies are expected to reduce the need for inpatient SUD hospitalization.

### 4.5 Prophylactic Tocilizumab

The reasons behind the initial lack of consensus in panel Rounds 1 and 2 for prophylactic tocilizumab statements are not clear. This approach reduces the incidence and severity of CRS [29, 30, 31, 32, 33, 34] but tocilizumab use is off‐label, access is not universal, some treatment centers may not have the necessary capabilities to administer it, and cost may be a factor (with some academic hospitals regularly having to absorb the cost). Based on premedication recommendations for CD3×CD20 BsAbs [35], and CRS treatment guidelines [3], dexamethasone has been explored prophylactically with BCMA‐BsAbs [36, 37, 38]. Although routine prophylactic (and less costly) dexamethasone is associated with reduced CRS severity, it did not reduce the incidence in all studies [37]. The final wording of Statements 72 and 73 reflected the heterogeneity of healthcare systems by stating that tocilizumab prophylaxis could be considered, regardless of hospital/outpatient setting, where appropriate and available.

### 4.6 Logistics

The consensus recommendations should also be considered in the context of logistical factors. This includes the number of doses during SUD (two for elranatamab, three for teclistamab), with 48‐h monitoring after each dose [4, 5]. These recommendations are based on the summary of product characteristics for the two drugs. The same drugs’ US package inserts indicate that patients should be hospitalized (for 48 and 24 h after the first and second step‐up doses of elranatamab, respectively, and for 48 h after each of the three step‐up doses for teclistamab) [39, 40]. However, we note that US experts are exploring outpatient SUD [26, 37]. In addition, although elranatamab dosing is fixed, teclistamab dosing is weight‐based and should be calculated before each dose [4, 5]. Fixed dosing has several practical advantages, including ease of preparation and administration, reduced cost, and lower risk of dosing errors [41, 42]. Combined, these logistical factors may impact patient/physician preference of BCMA‐BsAbs.

### 4.7 Strengths and Limitations

The implementation of these consensus recommendations has several potential benefits. First, they provide a harmonized framework for healthcare systems that currently vary in their approach to BCMA‐BsAb delivery for MM. Second, by defining minimum infrastructure requirements, they establish clear benchmarks for community centers and reduce uncertainty for clinicians by translating trial protocols into real‐world practice. Third, the consensus recommendations promote localizing care for the patient, thus creating greater access, particularly in regions where centralized delivery models may create geographic disparities (e.g., where travel to academic centers is prohibitive for patients without caregivers or flexible schedules). Since the demand for BCMA‐BsAbs will increase as they move to earlier lines of treatment [43, 44, 45, 46], educating on safe outpatient SUD and empowering the community to prescribe these treatments may offset additional resource utilization (e.g., hospital beds). This increased demand—and resulting patient load—will compete with chimeric antigen receptor T‐cell treatments for resources in academic settings (after the > 1‐month manufacturing period, infusions are typically done in academic settings, with 10–14 days of monitoring). Thus, outpatient SUD can increase and accelerate patient access to BCMA‐targeted therapies.

This consensus has several strengths, including methodological rigor and transparent reporting. A modified Delphi approach was used with a prespecified consensus threshold (≥ 75%), three survey rounds, adherence to ACCORD reporting guidance, and proactive seeking of additional German responses to improve representation. A multidisciplinary and multicountry panel was involved, with representation from physicians, nurses, and pharmacists based in France, Germany, Italy, Spain, Portugal, and the United Kingdom. In addition, the panel included representatives from community hospitals from all countries except Portugal, where hospitals are differentiated by type. These strengths help to expand upon guidance that is local (single country), is not based on Delphi panels, has fewer recommendations, or does not have as many representatives [14, 47].

This consensus is subject to limitations inherent in the Delphi methodology. While expert opinion provides valuable guidance where evidence is limited, it cannot substitute for prospective, real‐world data. The panel composition, although geographically diverse, may not fully reflect practice variation across Europe. Furthermore, as the countries involved were all classified as high income, applicability to low‐ and middle‐income regions may be limited. Finally, although product agnostic, these recommendations are tailored to subcutaneous BCMA‐BsAbs given the timing of their approvals. The applicability of these recommendations to intravenous agents or those targeting G protein–coupled receptor class C group 5‐member D requires further exploration and data generation. We envision the recommendations presented here may provide a robust foundation for similar efforts in non‐BCMA‐targeting BsAbs and intravenous BCMA‐BsAbs.

## 5 Conclusion

This Delphi consensus provides a pragmatic European framework for outpatient SUD and the transition of BCMA‐BsAb administration for MM from large university/academic hospitals or tertiary centers to small community centers. By establishing practical criteria for patients, centers, and processes, the consensus addresses critical safety concerns while promoting greater accessibility. It represents an important step toward integrating BCMA‐BsAbs into routine care and should serve as a foundation for future real‐world validation and guideline development.

## Author Contributions

All authors were involved in the survey conception/design or the acquisition, analysis, or interpretation of data. All authors contributed to the drafting of the manuscript and approved the final version.

## Funding

The study was initiated and funded by Pfizer. All co‐chairs, steering committee members, and panel members received honoraria from Pfizer as compensation for their time in contributing to this process. Triducive Partners Ltd. facilitated the project, analyzed the responses to the consensus statements in line with the Delphi methodology, and created the first draft of the manuscript; these activities were funded by Pfizer. Additional medical writing and editorial support was provided by Robyn Roth, PhD, of Nucleus Global and funded by Pfizer. The manuscript content and decision to submit for publication were controlled independently by the authors.

## Ethics Statement

A statement of consent was included at the start of the survey, and consent was implied by completion of the survey. Ethical approval was not required for this study, as it involved a noninterventional Delphi process with healthcare professionals only and did not involve patients, vulnerable populations, or the collection of sensitive or personally identifiable data. This study was not prospectively registered.

## Consent

The authors have nothing to report.

## Conflicts of Interest

María‐Victoria Mateos reports consultancy with and honoraria from AbbVie, Amgen, AstraZeneca, BMS/Celgene, GSK, J&J, Kite, Oncopeptides, Pfizer, Sanofi, and Menarini Stemline. Elena Zamagni reports consultancy with and honoraria from GSK, Amgen, BMS, Janssen, Menarini Stemline, Oncopeptides, Pfizer, and Sanofi. Massimo Gentile reports honoraria from AbbVie, AstraZeneca, BMS/Celgene, GSK, J&J, Beone, Pfizer, Sanofi, and Menarini Stemline. Mónica López Riñon reports honoraria from J&J, Amgen, Sanofi, BMS/Celgene, Abbvie, GSK. Florence Lachenal reports honoraria from BMS/Celgene, Gilead, Janssen, Roche, Pfizer and Sanofi. Rakesh Popat reports honoraria from AbbVie, BMS, GSK, J&J, Pfizer, Roche, Sanofi, and research funding from GSK and Pfizer. Christof Scheid reports consultancy with Amgen, BMS/Celgene, GSK, Janssen, Roche, Sanofi‐Aventis, honoraria from Amgen, BMS/Celgene, GSK, Janssen, Novartis, Oncopeptides, Pfizer, Sanofi‐Aventis, Stemline Therapeutics, Takeda, and research funding from Janssen and Takeda. Thomas Wolff reports research funding and honoraria from AbbVie, Bayer, Celgene, Novartis, Roche, and Teva. Isabel Perez‐Cruz reports employment by and stock options in Pfizer. Mohamad Mohty reports honoraria from Adaptive Biotechnologies, Amgen, Astellas, BMS/Celgene, Gilead, GSK, Janssen, Jazz Pharmaceuticals, MaaT Pharma, Medac Pharma, Novartis, Oncopeptides, Pfizer, Sanofi, Takeda, and Therakos. Rachel Hall declares no conflicts of interest.

## Supporting information




**Supporting File**: jha270290‐sup‐0001‐SuppMat.docx

## Data Availability

Upon request and subject to review, Pfizer will provide the data that support the findings of this study. Subject to certain criteria, conditions, and exceptions, Pfizer may also provide access to the related individual de‐identified data. See https://www.pfizer.com/science/clinical‐trials/trial‐data‐and‐results for more information.
