# Supplementary material for: European Recommendations for Transitioning the Care of Patients With Multiple Myeloma Treated With B‐Cell Maturation Antigen Bispecific Antibodies From Academic Hospitals to Community‐Based Centers and for Outpatient Step‐Up Dosing
Source: EJHaem. 2026 May 14;7(3):e70290. doi: 10.1002/jha2.70290 (PMC13172755; doi:10.1002/jha2.70290)
Supplement: Supplementary file 1 — Supporting File: jha270290‐sup‐0001‐SuppMat.docx [file JHA2-7-e70290-s001.docx]

**Supplementary Table 1. Round 1 statements and corresponding agreement**

| **No.** | **Statement** | **Agreement** |
| --- | --- | --- |
| 1 | Patients must be afebrile (≥24 hours) and hemodynamically stable before being considered for transition to community care. | 97% |
| 2 | Patients must be ASTCT Grade 0 (no toxicity) before being considered for transition to community care. | 89% |
| 3 | Patients must demonstrate stable hematologic, renal, and hepatic function, with no new or worsening cytopenias (e.g., neutropenia, thrombocytopenia), renal impairment, or transaminitis. | 89% |
| 4 | Patients should not be transitioned unless they have completed the full SUD phase and any occurring CRS or ICANS has resolved, except if they have not had CRS or ICANS with the first two doses. | 89% |
| 5 | Community centers delivering BCMA-BsAbs should be trained by clinical leads and resourced to manage CRS and ICANS using ASTCT and IMWG criteria. | 97% |
| 6 | Community centers delivering BCMA-BsAbs should have documented escalation protocols outlining how to formally contact the academic center, including procedures for 24-hour communication, in case support or intervention is needed. | 89% |
| 7 | Community centers delivering BCMA-BsAbs should have on-site access to routine urgent blood tests (e.g., FBC, CRP, LFT, renal function). | 97% |
| 8 | Community centers delivering BCMA-BsAbs should have 24/7 access to tocilizumab and corticosteroids (e.g., dexamethasone) for rapid CRS or ICANS intervention and trained personnel to administer. | 95% |
| 9 | Community centers delivering BCMA-BsAbs should be located within 60 minutes (by ambulance, car, etc.) of a facility with access to inpatient facilities. | 97% |
| 10 | All patients should undergo baseline assessment for CMV (serology followed by PCR if serology is positive for CMV) to allow for accurate assessment of subsequent reactivation. | 92% |
| 11 | Patients with immunoparesis irrespective of the target IgG should receive IV/SC Ig, particularly if there is a risk of recurrent or severe infections. | 84% |
| 12 | Patients with trough IgG <400 mg/dL should receive IV or SC Ig every 4 weeks and be retested every month until IgG recovers (including after completion of treatment). | 95% |
| 13 | Where possible, neutropenia (defined as an absolute neutrophil count <1000/mm^3^) should be resolved, using G-CSF as needed, prior to SUD. | 95% |
| 14 | Baseline and monitoring of serum immunoglobulin levels (IgG) every 4 weeks should be conducted, with timely access to IV/SC Ig as clinically indicated. | 100% |
| 15 | Subcutaneous Ig may be considered if available to reduce hospital administrative burden while maintaining immunoprophylaxis. | 95% |
| 16 | Antiviral (e.g., acyclovir) and *PJP* prophylaxis (e.g., co-trimoxazole) is mandatory for all patients. | 100% |
| 17 | Antifungal (e.g., posaconazole/fluconazole) and antibacterial prescriptions should be tailored to individual risk factors, prior lines of therapy, and lymphodepletion status. | 100% |
| 18 | Academic centers should provide 3–5 days’ notice before transitioning care to the community center. | 84% |
| 19 | A comprehensive treatment summary, including BsAb dosing history, CRS/ICANS grading, and infection prophylaxis, and all AEs and management must be provided at handover. | 100% |
| 20 | Patients and care partners must receive education on early signs/symptoms of CRS, ICANS, and infection (e.g., fever, confusion, respiratory symptoms) at discharge. | 100% |
| 21 | Follow-up with academic centers via telemedicine, a virtual meeting, or a phone call should be scheduled within 1 week of transition. | 76% |
| 22 | Patients and care partners should receive a 24/7 emergency contact and escalation plan before transition. | 97% |
| 23 | Education on CRS/ICANS must include both verbal explanations and written materials tailored to BCMA-BsAb toxicities. | 100% |
| 24 | Language-appropriate educational materials should be provided to patients and care partners in their first language, where possible. | 100% |
| 25 | Outpatient SUD should only be considered for patients with ECOG performance status of 0, 1, or 2. | 92% |
| 26 | Outpatient SUD should only be considered for patients with low tumor burden (where high tumor burden is defined as BMPCs ≥60%, presence of ≥1 focal lesion ≥5 mm [bone or soft tissue] on MRI or PET-CT, M‑spike ≥30 g/L, LDH > ULN) and stable disease status. | 84% |
| 27 | Outpatient SUD should only be considered for patients with lytic bone lesions if potential complications (e.g., fracture risk, spinal cord compression) have been evaluated and appropriate monitoring and support measures are in place. | 87% |
| 28 | Outpatient SUD should only be considered for patients with renal impairment or failure, including chronic kidney disease at any stage, if renal function is stable enough to support safe outpatient management. | 87% |
| 29 | Outpatient SUD should only be considered for patients with ESRD providing they are clinically stable and have reliable access to dialysis, and structured pathways are in place for toxicity monitoring and escalation. | 82% |
| 30 | Outpatient SUD should only be considered for patients with hypertension if it is well controlled and not associated with additional cardiovascular risks. | 100% |
| 31 | Outpatient SUD should only be considered for patients with congestive heart failure if they are clinically stable (e.g., NYHA class I/II), have no recent decompensation, and have undergone appropriate cardiac evaluation. | 95% |
| 32 | Outpatient SUD should only be considered for patients with chronic pulmonary disease if respiratory status is stable and oxygenation needs are met (e.g., FEV₁ >60%). | 95% |
| 33 | Outpatient SUD should only be considered for patients with a history of myocardial infarction if they have preserved cardiac function (LVEF of ≥45%), are clinically stable, and are not at high risk for further cardiovascular events. | 100% |
| 34 | Outpatient SUD should only be considered for patients with Grade 3/4 neutropenia if it is appropriately managed with prophylactic measures and close monitoring. | 84% |
| 35 | Outpatient SUD should only be considered for patients with no neurological symptoms (e.g., cognitive impairment, focal neurological deficits, or seizure risk factors). | 100% |
| 36 | Outpatient SUD should only be considered for patients with cerebrovascular disease if they are neurologically stable and not at high risk for acute events. | 89% |
| 37 | Outpatient SUD should only be considered for patients with peripheral vascular disease if it is not associated with high thrombotic risk during treatment. | 89% |
| 38 | Outpatient SUD should only be considered for patients with diabetes if it is well managed and without acute complications. | 95% |
| 39 | Outpatient SUD should only be considered for patients with hypercalcemia if it is corrected and stable before SUD initiation. | 92% |
| 40 | Outpatient SUD should only be considered for patients with no active infection (e.g., CMV reactivation, upper respiratory tract infection). | 97% |
| 41 | Patients must reside within 1 hour (by ambulance, car, etc.) of a treatment center that provides both hematology or internal medicine expertise and ICU-level care. | 100% |
| 42 | Patients must have a suitable adult care partner available 24/7 during the SUD period who has received CRS/ICANS training. | 100% |
| 43 | Patients must have a responsible adult care partner available 24/7 during the SUD period who is physically and cognitively able to support the patient and understands when to seek urgent care. | 100% |
| 44 | The center/provider that provided CRS/ICANS training for the care partner should be recorded by the trainer and a copy retained in the patient notes. | 95% |
| 45 | Outpatient SUD should only be delivered in centers that can coordinate access to an ICU. | 95% |
| 46 | Outpatient SUD centers must ensure availability of a clinician (e.g., hematologist or trained advanced clinical practitioner) experienced in managing CRS and ICANS during dosing hours (e.g., experienced in use of BsAbs and able to supervise outpatient SUD). | 97% |
| 47 | Outpatient SUD centers must have tocilizumab (two doses) available in a known and accessible location. | 97% |
| 48 | Outpatient SUD centers must have access to dexamethasone, high-dose corticosteroids, and anakinra for management of emergent CRS or ICANS. | 97% |
| 49 | Outpatient SUD centers must have access to oral valganciclovir or letermovir or alternatively intravenous ganciclovir or foscarnet for treatment of reactivated or emergent CMV. | 97% |
| 50 | SOPs must define observation duration and escalation thresholds for CRS and ICANS, using established grading systems such as ASTCT and ICE. | 100% |
| 51 | Emergency care must be accessible within 30–60 minutes of the outpatient SUD center and must provide fast-track access protocol, inpatient admission capability, access to intensive care, and direct communication with the treating hematology team. | 97% |
| 52 | Following each step-up dose, patients must be observed for vital signs and neurological function for a minimum defined period as specified in the SmPC. | 100% |
| 53 | Standardized admission protocols aligned with the SmPC are required for outpatient SUD. | 100% |
| 54 | Biological high-risk disease can be defined as the presence of ≥1 of the following:   1. At least 1 of (a) del(17p), with a cutoff of >20% clonal fraction, and/or TP53 mutation; (b) an IgH translocation including t(4;14), t(14;16), or t(14;20) along with 1q+ and/or del(1p32); (c) monoallelic del(1p32) along with 1q+ or biallelic del(1p32); or (d) β_2_-microglobulin ≥5.5 mg/L with normal creatinine (<1.2 mg/dL)  2. Extramedullary plasmacytomas  3. Plasma cell leukemia  4. High-risk gene expression profiling signature | 100% |
| 55 | Clinical high-risk disease can be defined as ≥1 of the following: 1. Cardiovascular diseases (especially heart failure); 2. Poorly controlled diabetes; 3. COPD; 4. Severe chronic kidney disease; 5. Dementia. | 89% |
| 56 | CMV infection/reactivation should not be routinely monitored during SUD unless febrile illness occurs without bacterial cause. | 82% |
| 57 | In suspected CMV infection/reactivation, BsAb therapy should be placed on hold, with active monitoring of the viral load. | 95% |
| 58 | Premedications (e.g., dexamethasone, antihistamines, paracetamol) must be administered before each step-up dose in accordance with the SmPC. | 97% |
| 59 | Neurotoxicity should be assessed and documented using the ICE score during SUD and at each subsequent visit. | 95% |
| 60 | CRS should be assessed and documented using ASTCT criteria. | 100% |
| 61 | Remote monitoring tools (e.g., check-in calls) may be useful to support patients during outpatient SUD. | 100% |
| 62 | Protocols should be established to enable immediate admission to the treating center for prompt management of toxicities. | 100% |
| 63 | Prophylactic tocilizumab may be considered prior to the first step-up dose in patients to reduce the risk of CRS, if available and according to prescriber discretion. | 66% |
| 64 | Oral dexamethasone (10–20 mg) can be used only under direct instruction from a treating physician when CRS or ICANS is suspected, en route to the hospital, or as a strategy for at-home management of Grade 1 CRS or ICANS. Clear criteria and escalation instructions must be provided to patients and care partners along with education regarding the risks of inappropriate use. | 92% |
| 65 | A minimum interval of 48 hours must be maintained between step-up doses, as per the SmPC. | 100% |
| 66 | Given the typical onset of CRS/ICANS within 1–3 days of dosing, patients should undergo a clinical review, either in person or virtually, within 24–72 hours of completing the SUD schedule. | 100% |
| 67 | Outpatients should have a normal ICE score before leaving the treatment facility following the final step-up dose. | 100% |
| 68 | All clinical and pharmacy staff supporting SUD should be trained in CRS/ICANS and infection recognition (including opportunistic), grading, and emergency management. | 100% |
| 69 | Patients and care partners should receive clear verbal and written instructions on recognizing complications and how to access urgent care pathways. | 97% |

AE: adverse event; ASTCT: American Society for Transplantation and Cellular Therapy; BCMA: B-cell maturation antigen; BMPC: bone marrow plasma cell; BsAb: bispecific antibody; CMV: cytomegalovirus; COPD: chronic obstructive pulmonary disease; CRP: C-reactive protein; CRS: cytokine release syndrome; ECOG: Eastern Cooperative Oncology Group; ESRD: end-stage renal disease; FEV_1_: forced expiratory volume in 1 second; FBC: full blood count; G-CSF: granulocyte colony-stimulating factor; ICANS: immune effector cell–associated neurotoxicity syndrome; ICE: immune effector cell–associated encephalopathy; ICU: intensive care unit; Ig: immunoglobulin; IgH: immunoglobulin heavy chain; IMWG: International Myeloma Working Group; IV: intravenous; LDH: lactate dehydrogenase; LFT: liver function test; LVEF: left ventricular ejection fraction; MRI: magnetic resonance imaging; NYHA: New York Heart Association; PET-CT: positron emission tomography–computed tomography; PCR: polymerase chain reaction; PJP: Pneumocystis jirovecii pneumonia; SC: subcutaneous; SmPC: summary of product characteristics; SOP: standard operating procedure; SUD: step-up dosing; TP53: tumor protein p53; ULN: upper limit of normal.

**Supplementary Table 2. Round 2 statements and corresponding agreement**

| **No.** | **Statement** | **Agreement** |
| --- | --- | --- |
| 1 | Patients must be afebrile (≥24 hours) and hemodynamically stable before being considered for transition to community care. | 97% |
| 2 | Patients must be ASTCT Grade 0 (no toxicity) for CRS and ICANS before being considered for transition to community care. | 95% |
| 3 | Prophylactic IV/SC Ig prior to the first dose of BsAb therapy in multiple myeloma should be considered on a case-by-case basis for all patients (e.g., taking IgG levels and BsAb type into account). | 82% |
| 4 | Prophylactic IV/SC Ig prior to the first dose of BsAb therapy in multiple myeloma should be considered for all patients with IgG <400 mg/dL. | 95% |
| 5 | Prophylactic IV/SC Ig prior to the first dose of BsAb therapy in multiple myeloma should be considered for all patients with recurrent infections. | 89% |
| 6 | Prophylactic IV/SC Ig should be initiated early and maintained in patients with IgG <400 mg/dL or with recurrent infections. | 97% |
| 7 | Neutropenia (defined as an absolute neutrophil count <1000/mm^3^) should be resolved, using G-CSF, prior to SUD. | 87% |
| 8 | Neutropenia (defined as an absolute neutrophil count <1000/mm³) should not be resolved with G-CSF prior to SUD ***if infiltrative***, due to the risk of CRS. | 66% |
| 9 | Language-appropriate educational materials must be provided to patients and care partners in their first language. | 100% |
| 10 | Outpatient SUD should only be considered for patients with chronic pulmonary disease if respiratory status is stable and oxygenation needs are met (e.g., FEV₁ >50%). | 89% |
| 11 | Outpatient SUD should generally not be offered to patients with Grade 3/4 neutropenia unless in exceptional, carefully monitored settings. A minimum ANC ≥1000/mm³ is generally recommended. | 95% |
| 12 | Patients must reside within 60 minutes (by ambulance, car, etc.) of a treatment center that provides both hematology or internal medicine expertise and ICU-level care. | 100% |
| 13 | The center/provider that provided CRS/ICANS training for the care partner should be documented by the trainer and a copy retained in the patient notes, if required. | 87% |
| 14 | A formal protocol should be developed to guide reescalation of care to the academic center for patients experiencing clinical deterioration following transfer to a community site after BsAb therapy initiation. This protocol should integrate rapid-response triggers based on clinical efficacy, safety indicators, and/or patient-centered factors (e.g., quality of life, goals of care), with flexibility for urgent transfers and adaptation to local resources. | 92% |
| 15 | Biological high-risk disease can be defined as the presence of ≥1 of the following: | 95% |
| 15a | At least 1 of (a) del(17p), with a cutoff of >20% clonal fraction, and/or TP53 mutation; (b) an IgH translocation including t(4;14), t(14;16), or t(14;20) along with 1q+ and/or del(1p32); (c) monoallelic del(1p32) along with 1q+ or biallelic del(1p32); or (d) β_2_-microglobulin ≥5.5 mg/L with normal creatinine (<1.2 mg/dL). | 92% |
| 15b | ISS Stage III disease | 89% |
| 15c | Elevated LDH ≥223 U/L | 97% |
| 15d | Extramedullary plasmacytomas | 97% |
| 15e | Plasma cell leukemia | 89% |
| 16 | Systematic AE reporting processes using standardized criteria (e.g., CTCAE) should be in place for all patients receiving BsAbs, regardless of the treatment setting, and should be simple, practical, and integrated into routine workflows. | 95% |
| 17 | Neurotoxicity should be assessed and documented using both ICE and ASTCT criteria during SUD and at each subsequent visit. | 97% |
| 18 | Toxicity data should be collected through the first full treatment dose and beyond 14 days. | 95% |
| 19 | CRS should be assessed and documented using ASTCT CRS criteria. | 100% |
| 20 | Patients undergoing SUD should receive remote daily safety calls from a member of the medical team, except on days when they are seen in person at the hospital or clinic. | 92% |
| 21 | Prophylactic tocilizumab is recommended prior to the first step-up dose in patients to reduce the risk of CRS, where appropriate and available. | 66% |
| 22 | A single IV 8-mg/kg dose of tocilizumab (max dose of 800 mg) should be administered as a pretreatment on C1D1, 1 to 2 hours prior to starting administration of BCMA-BsAb therapy for outpatient SUD, where appropriate and available. | 66% |
| 23 | In the absence of tocilizumab prophylaxis, prophylactic dexamethasone (12 mg) should be considered on D2 of SUD. | 76% |
| 24 | In the absence of tocilizumab prophylaxis, prophylactic dexamethasone (12 mg) should be considered on D4 of SUD. | 74% |
| 25 | All clinical and pharmacy staff supporting SUD should be trained in CRS/ICANS and infection recognition (including opportunistic), grading, and emergency management. | 100% |
| 26 | Staff at community centers administering BsAb therapy should undergo simple, practical, structured training (e.g., using quick-reference materials), as defined by a standardized protocol. This training should cover key topics such as CRS/ICANS management, patient monitoring, and emergency escalation procedures, and completion documented appropriately. | 100% |

AE: adverse event; ANC: absolute neutrophil count; ASTCT: American Society for Transplantation and Cellular Therapy; BCMA: B-cell maturation antigen; BsAb: bispecific antibody; C: cycle; CRS: cytokine release syndrome; CTCAE: Common Terminology Criteria for Adverse Events; D: day; FEV_1_: forced expiratory volume in 1 second; G-CSF: granulocyte colony-stimulating factor; ICANS: immune effector cell–associated neurotoxicity syndrome; ICE: immune effector cell–associated encephalopathy; ICU: intensive care unit; Ig: immunoglobulin; IgH: immunoglobulin heavy chain; ISS: International Staging System; IV: intravenous; LDH: lactate dehydrogenase; SC: subcutaneous; SUD: step-up dosing; TP53: tumor protein p53.

**Checklists**

**Outpatient BsAb (MM) Consensus Checklists**

Use locally and adapt as needed.

**A. Transition of Care From Academic to Community Settings**

**A1. Patient readiness**

| Criteria | Criteria met |
| --- | --- |
| Afebrile for ≥24 hours | ☐ |
| ASTCT Grade 0 toxicity at time of transfer | ☐ |
| Stable hematologic, renal, and hepatic function (no new/worsening cytopenias, renal impairment, or transaminitis) | ☐ |
| Completed full SUD and any CRS/ICANS resolved (or no CRS/ICANS with first two doses) | ☐ |

ASTCT: American Society for Transplantation and Cellular Therapy; CRS: cytokine release syndrome; ICANS: immune effector cell–associated neurotoxicity syndrome; SUD: step-up dosing.

**A2. Community center readiness**

| Criteria | Criteria met |
| --- | --- |
| Staff trained to recognize/grade/manage CRS and ICANS | ☐ |
| Documented 24/7 escalation pathways to academic center | ☐ |
| On‑site access to urgent labs (FBC, CRP, LFT, renal function) | ☐ |
| 24/7 access to tocilizumab and corticosteroids; trained personnel to administer | ☐ |
| Located within ~60 minutes of facility with inpatient and ICU capability | ☐ |

CRP: C-reactive protein; CRS: cytokine release syndrome; FBC: full blood count; ICANS: immune effector cell–associated neurotoxicity syndrome; ICU: intensive care unit; LFT: liver function test.

**A3. Infection and immunoglobulin management**

| Criteria | Criteria met |
| --- | --- |
| Baseline CMV serology (PCR if seropositive) capability | ☐ |
| Antiviral (e.g., acyclovir) and *PJP* prophylaxis capability | ☐ |
| Antifungal/antibacterial prophylaxis capability | ☐ |
| Immunoglobulin monitoring capability in place  (at baseline and every 4 weeks during therapy) | ☐ |
| IV/SC Ig administration capability in place | ☐ |

CMV: cytomegalovirus; Ig: immunoglobulin; IV: intravenous; LFT: liver function test; PCR: polymerase chain reaction; PJP: Pneumocystis jirovecii pneumonia; SC: subcutaneous.

**A4. Handover and documentation**

| Criteria | Criteria met |
| --- | --- |
| Agreement in place for academic site to provide 3–5 days’ notice of transition | ☐ |
| Methods of communication and key contact details provided for each site | ☐ |
| Format of treatment summary agreed upon between sites (doses; CRS/ICANS grading/management; infection prophylaxis; AEs) | ☐ |
| Scheduling mechanism for follow‑up call/telemedicine with academic team in place | ☐ |

AE: adverse event; CRS: cytokine release syndrome; ICANS: immune effector cell–associated neurotoxicity syndrome.

**A5. Patient education and support**

| Criteria | Criteria met |
| --- | --- |
| 24/7 emergency contact number and clear escalation plan materials selected/developed and available | ☐ |
| Written materials on CRS/ICANS/infection red flags selected/developed (fever, confusion, dyspnea) | ☐ |
| All developed materials adapted to reflect local needs of patients and care partners | ☐ |

CRS: cytokine release syndrome; ICANS: immune effector cell–associated neurotoxicity syndrome.

**B. Outpatient Step-Up Dosing**

**B1. Patient eligibility**

| Criteria | Yes | Not applicable |
| --- | --- | --- |
| ECOG performance status of 0–2 | ☐ |  |
| Low tumor burden (e.g., BMPCs <60%, no ≥5-mm focal lesion on MRI/PET-CT, M‑spike <30 g/L, LDH ≤ ULN) | ☐ |  |
| Lytic lesions assessed and support/monitoring plans in place | ☐ | ☐ |
| Renal impairment/kidney failure: clinically stable with reliable dialysis access | ☐ | ☐ |
| Hypertension well controlled; CHF stable (e.g., NYHA class I/II) | ☐ | ☐ |
| Chronic pulmonary disease stable (e.g., FEV_1_ >50%) | ☐ | ☐ |
| History of MI and clinically stable with preserved LVEF ≥45% | ☐ | ☐ |
| ANC ≥1000/mm³ (Grade 3/4 neutropenia considered ineligible for outpatient SUD except in exceptional, closely monitored cases) | ☐ |  |
| No neurological symptoms (cognitive deficits, focal deficits, seizure risk) | ☐ |  |
| CVA/PVD stable | ☐ | ☐ |
| Diabetes well managed | ☐ | ☐ |
| Corrected/stable hypercalcemia | ☐ | ☐ |
| No active infection (e.g., CMV reactivation, URTI) | ☐ | ☐ |
| Residence within ~60 minutes of a center with hematology/internal medicine expertise and ICU access | ☐ |  |
| Care partner available 24/7 during SUD | ☐ |  |
| Care partner trained in CRS/ICANS red flags and training documented in notes | ☐ |  |

ANC: absolute neutrophil count; BMPC: bone marrow plasma cell; CHF: congestive heart failure; CMV: cytomegalovirus; CRS: cytokine release syndrome; CVA: cerebrovascular accident; ECOG: Eastern Cooperative Oncology Group; FEV_1_: forced expiratory volume in 1 second; ICANS: immune effector cell–associated neurotoxicity syndrome; ICU: intensive care unit; LDH: lactate dehydrogenase; LVEF: left ventricular ejection fraction; MI: myocardial infarction; MRI: magnetic resonance imaging; NYHA: New York Heart Association; PET-CT: positron emission tomography–computed tomography; PVD: peripheral vascular disease; SUD: step-up dosing; ULN: upper limit of normal; URTI: upper respiratory tract infection.

**B2. Center criteria and protocols**

| Criteria | Criteria met |
| --- | --- |
| Agreed-upon communication mechanisms between the center and ICU and emergency services | ☐ |
| SOP includes requirement of experienced clinician (BsAb/CRS/ICANS expertise) available during dosing hours | ☐ |
| Tocilizumab available and in an accessible location | ☐ |
| Dexamethasone/high‑dose steroids/anakinra available for use | ☐ |
| Valganciclovir/letermovir (or IV ganciclovir/foscarnet) available | ☐ |
| SOPs define observation times and escalation thresholds using ASTCT CRS/ICE scores | ☐ |
| Emergency care accessible within 30–60 minutes | ☐ |
| Fast‑track access to emergency care | ☐ |
| Observation schedules (in line with SmPC) defined in SOP | ☐ |
| Formal protocol in place for reescalation to academic center | ☐ |
| Antihistamine and paracetamol available for use | ☐ |
| Neurotoxicity and CRS assessed and documented using ASTCT/ICE criteria at SUD and each visit | ☐ |
| Collect toxicity data through first full treatment dose and ≥14 days thereafter | ☐ |

ASTCT: American Society for Transplantation and Cellular Therapy; BsAb: bispecific antibody; CRS: cytokine release syndrome; ICANS: immune effector cell–associated neurotoxicity syndrome; ICE: immune effector cell–associated encephalopathy; ICU: intensive care unit; IV: intravenous; SmPC: summary of product characteristics; SOP: standard operating procedure; SUD: step-up dosing.

**B3. Defining high-risk patients (for triage)**

| Criteria | Yes | No |
| --- | --- | --- |
| **Biological high risk** |  |  |
| del(17p) (>20% clone) and/or TP53 mutation; t(4;14)/t(14;16)/t(14;20) ± 1q+ and/or del(1p32); monoallelic del(1p32) + 1q+ or biallelic del(1p32) | ☐ | ☐ |
| β_2_‑microglobulin ≥5.5 mg/L with normal creatinine | ☐ | ☐ |
| ISS Stage III disease |  |  |
| Elevated LDH (≥223 U/L) | ☐ | ☐ |
| Extramedullary disease | ☐ | ☐ |
| Plasma cell leukemia | ☐ | ☐ |
| Validated high‑risk GEP | ☐ | ☐ |
| **Clinical high risk** |  |  |
| Heart failure | ☐ | ☐ |
| Poorly controlled diabetes | ☐ | ☐ |
| COPD | ☐ | ☐ |
| Severe CKD/kidney failure | ☐ | ☐ |
| Dementia | ☐ | ☐ |

CKD: chronic kidney disease; COPD: chronic obstructive pulmonary disease; GEP: gene expression profile; ISS: International Staging System; LDH: lactate dehydrogenase; TP53: tumor protein p53.

**B4. Logistics and follow-up**

| Criteria | Yes |
| --- | --- |
| SOPs specify the following: |  |
| Appropriate (≥48‑hour) interval between step-up doses (per relevant SmPC) | ☐ |
| Clinical review methods (in person or virtual) and timings (within 24–72 hours after completing SUD) | ☐ |
| Requirement for normal ICE score documented before discharge after final step‑up dose | ☐ |
| Remote daily safety calls on nonvisit days during SUD | ☐ |
| Immediate admission protocols | ☐ |

ICE: immune effector cell–associated encephalopathy; SmPC: summary of product characteristics; SOP: standard operating procedure; SUD: step-up dosing.

**Note:** These checklists are based on the developed consensus statements and do not replace the summary of product characteristics, national guidance, or institutional SOPs.
